# Li_7_La_3_Zr_2_O_12_ Garnet Solid Polymer Electrolyte for Highly Stable All-Solid-State Batteries

**DOI:** 10.3389/fchem.2020.619832

**Published:** 2021-01-18

**Authors:** Quoc Hung Nguyen, Van Tung Luu, Hoang Long Nguyen, Young-Woo Lee, Younghyun Cho, Se Young Kim, Yun-Seok Jun, Wook Ahn

**Affiliations:** ^1^Department of Energy Systems Engineering, Soonchunhyang University, Asan-si, South Korea; ^2^Department of Chemistry, University of Waterloo, Waterloo, ON, Canada; ^3^Microcellular Plastics Manufacturing Laboratory, Department of Mechanical and Industrial Engineering, University of Toronto, Toronto, ON, Canada

**Keywords:** all-solid-state batteries, cubic garnet LLZO, ionic-liquid, solid polymer electrolyte, lithium dendrite growth suppression

## Abstract

All-solid-state batteries have gained significant attention as promising candidates to replace liquid electrolytes in lithium-ion batteries for high safety, energy storage performance, and stability under elevated temperature conditions. However, the low ionic conductivity and unsuitability of lithium metal in solid polymer electrolytes is a critical problem. To resolve this, we used a cubic garnet oxide electrolyte (Li_7_La_3_Zr_2_O_12_ – LLZO) and ionic liquid in combination with a polymer electrolyte to produce a composite electrolyte membrane. By applying a solid polymer electrolyte on symmetric stainless steel, the composite electrolyte membrane shows high ionic conductivity at elevated temperatures. The effect of LLZO in suppressing lithium dendrite growth within the composite electrolyte was confirmed through symmetric lithium stripping/plating tests under various current densities showing small polarization voltages. The full cell with lithium iron phosphate as the cathode active material achieved a highest specific capacity of 137.4 mAh g^−1^ and a high capacity retention of 98.47% after 100 cycles at a current density of 50 mA g^−1^ and a temperature of 60°C. Moreover, the specific discharge capacities were 137 and 100.8 mAh g^−1^ at current densities of 100 and 200 mA g^−1^, respectively. This research highlights the capability of solid polymer electrolytes to suppress the evolution of lithium dendrites and enhance the performance of all-solid-state batteries.

## Introduction

Lithium-ion batteries (LIBs) currently play a principal role in energy storage technologies, having a wide range of applications from automotive vehicles to electronic devices, owing to their high energy density, ecological friendliness, fast charging time, and long cycle life (Abdin and Khalilpour, 2019). Compared to other materials, lithium is characterized by the uppermost theoretical capacity (3,860 mAh g^−1^), low density, and the minimum redox potential (3.04 V vs. standard hydrogen electrode) (Ahn et al., 2019; Wang et al., 2020). In addition, LIBs show good performance for application in electric vehicles (EVs). However, due to the superior reactivity of lithium metal, the continuous irregular electrochemical stripping and coating of lithium ions consumes a large amount of liquid electrolyte during cycling. This produces lithium dendrites. As the cycling continues, the growing lithium dendrites pierce the separator, triggering a short circuit. Furthermore, the ohmic heat produced by this phenomenon can cause thermal runaway and disastrous battery failure (Finegan et al., 2017; Cao et al., 2020; Xiong et al., 2020).

It has been reported that the dendrite formation can be mitigated, theoretically, by changing the electric field distribution homogeneously, promoting Li ion diffusion, and by mechanical blocking (Tikekar et al., 2016; Pang et al., 2017). Several research directions have been proposed to suppress the creation of lithium dendrites, prevent the dendrites from causing short-circuits within the battery, and improve the battery performance (Han et al., 2019; Song et al., 2019). These strategies are mainly investigated following the dendrite evolution process from flushing to nucleation and finally growth. Electrolyte modification using additives is one of the most promising methods to diminish dendrite evolution from the flushing stage (Xu et al., 2017). A small amount of additives could lead to the introduction of a stable solid electrolyte boundary, resulting in a homogeneous Li ion distribution and decrease in dead lithium formation (Ding et al., 2013; Cheng et al., 2016). Scaffolds with a homogeneous distribution of lithiophilic nucleation sites could lead to homogeneous Li ion flux and nucleation morphology, promoting even Li deposition and minimizing dendrite evolution (Liang et al., 2015). To prevent dendrites from puncturing the separator, a solid-state electrolyte with a high modulus could be the solution for establishing a safe Li metal battery cycling. Among these approaches, solid-state electrolytes such as solid electrolytes and solid polymer electrolytes are the most promising because of the drawbacks of liquid electrolytes in terms of toxicity and flammability. However, solid electrolytes show lower ionic conductivity and higher price compared to solid polymer electrolytes (Croce et al., 1998; Minami et al., 2006); therefore, solid polymer electrolytes are more likely to be considered.

To date, polyethylene oxide (PEO)-based materials still remain to be the promising polymer host candidates in solid polymer electrolytes (SPEs) because of their excellent permittivity, great Li ion solvating capability, and high chain flexibility (Mindemark et al., 2018; Kurzweil and Brandt, 2019). Moreover, the PEO offers several additional advantages, such as excellent electrochemical stability, ease of fabrication, low cost and improved safety (Jiang et al., 2018). However, ionic conductivities of typical linear PEO-based SPEs at room temperature are normally low (≤10^−6^ S cm^−1^) owing to the high crystallinity of the EO (Wang et al., 2018). To enhance ionic conductivities, strategies including the use of block copolymer electrolytes (Pelz et al., 2018), cross-linked polymer electrolytes (Sakakibara et al., 2019), interpenetrating network polymer electrolytes (Tong et al., 2018), and composite polymer electrolytes (Liu et al., 2017; Yao et al., 2019) have been proposed. Furthermore, SPEs are typically operated at a high temperature (usually at 60°C) to achieve good battery performance; however, under this condition, ionic conductivities can be low, and electrode/electrolyte interfacial resistances may increase. To address this issue, ionic liquids can be used as cross-links onto a PEO polymer matrix; this additive could improve the properties of SPEs, such as minimum volatilization, excellent thermal stability, extensive electrochemical stability window, and great ionic conductivity.

The oxide solid electrolyte Li_7_La_3_Zr_2_O_12_ (LLZO) is thought to be very convincing solid electrolyte materials because of its excellent wide-ranging performance, such as greater Li^+^ conductivity, broader electrochemical window (>5 V), and high stability in air (Buschmann et al., 2011; Fu et al., 2017; Li et al., 2019). More importantly, LLZO and ionic liquids can suppress the accumulation of lithium dendrites during cycling. The excellent electrochemical performance of LLZO suggests prospects of wider application in lithium metal batteries by applying the LLZO as a filler additive in the PEO polymer electrolyte (Buschmann et al., 2011; Li et al., 2018).

In this work, cubic phase LLZO was produced using the sol-gel method, which is a simple way to synthesize LLZO at low temperatures. By adding 10% of LLZO into the SPEs, the ionic conductivity doubled and the battery capacity was enhanced by 24% at 50 mA g^−1^ current density and 60°C compared to a cell with the electrolyte without LLZO. In particular, the battery with PLL electrolyte exhibited outstanding performance with a high specific capacity of 137.4 mAh g^−1^ at the maximal point and 135.3 mAh g^−1^ after 100 cycles with 98.47% capacity retention at 50 mA g^−1^ current density. Moreover, the specific discharge capacities were 137 and 100.8 mAh g^−1^ at 100 and 200 mA g^−1^ current density, respectively. The result is promising for the use of all-solid-state batteries in future applications.

## Experimental

### Synthesis of Cubic Garnet-Type LLZO Following the Sol-Gel Method

Cubic-type LLZO nanoparticle samples were synthesized *via* an easy one-step Pechini sol-gel method (Jin and McGinn, 2011, 2013; Tao et al., 2017). 0.0078 mol C_6_H_8_O_7_·H_2_O, 0.0077 mol LiNO_3_ (Sigma) (10% of Li was exsit), 0.003 mol of La(NO_3_)_3_·xH_2_O (Sigma), and 0.002 mol of ZrO(NO_3_)_2_·xH2O (Sigma) were dissolved in 10 mL deionized water to obtain a homogeneous solution under constant stirring and heating. After drying the sol at 250°C for 9.5 h, a brown porous precursor was hand-milled using a mortar and transferred to an alumina crucible boat to obtain a pure environment at high temperature. After the transfer, the brown precursor was calcined at various temperatures such as 600, 800, and 1,000°C for 6.5 h in air to obtain cubic phase Al-doped LLZO nanoparticles and demonstrate the effect of temperature on the LLZO sol-gel synthesis.

### Fabrication of the Electrolyte Membrane

All experiments were performed in glove boxes under argon atmosphere to minimize the influence of water and oxygen on the raw materials. The PEO-LiTFSI-10 wt% Pyr_1,3_TFSI-10 wt% LLZO (PLL) electrolyte solution was fabricated *via* a conventional solution casting technique. First, PEO (MW = 4 × 10^5^ g, Sigma) and LiTFSI (99%, Sigma) were dissolved in acetonitrile (ACN, 99%, Sigma) to obtain a homogeneous solution, where the molar ratio of PEO and LiTFSI was 18:1. Next, 10 wt% of ionic liquid Pyr_1,3_TFSI (TCI, 99%) was slowly added into the slurry, and 10% of LLZO powder was also added. The mixture was sonicated for 1 h using an ultrasonic machine at high power to improve the dispersion of LLZO. The homogeneous slurry was continuously stirred overnight inside the glove box. Subsequently, the electrolyte membrane was gained by wet coating the final slurry on a Teflon plate and drying at 45°C in a vacuum oven for 24 h to evaporate the acetonitrile. They were then stored in a glove box for 12 h before being used to assemble the cell for electrochemical analysis. PL (without LLZO) was also obtained using the same method. The thickness of the electrolyte membrane was controlled and measured using thickness gauges. The average thickness of the electrolyte membrane was ~100 μm.

## Characterizations

### Material Characterization

The crystal structures of LLZO powder at various temperatures were determined by X-ray diffraction (XRD, Rigaku) using Cu/Kα (λ = 0.15418 nm) measurement with a 2θ range of 10–90° and scan rate of 3°/min. The morphology images of the LLZO powder and electrolyte membrane were captured by scanning electron microscopy (SEM) on a JSM-6010LA fitted with an energy-dispersive X-ray spectrum (EDS) detector.

### Electrochemical Characterization of the Electrolyte

All the coin cells need at least 2 h for activation at 60°C to assure solid contact between the electrode and electrolyte. A ZIVE MP2 electrochemical workstation system (from WonA Tech ZIVE LAB) was utilized to measure and investigate the electrochemical characterization of the polymer electrolyte. The ionic conductivity at temperatures ranging from 25 to 80°C was obtained through electrochemical impedance spectroscopy (EIS) in the frequency range from 1 MHz to 1 Hz using a symmetric stainless steel electrode system (SS/electrolyte/SS). During the measurements, the cell should remain at each temperature for 1 h before EIS measurements because of the effect of temperature. The ionic conductivity of the electrolyte was calculated from the impedance and thickness of the electrolyte using the equation σ = L/R·A (where L is the electrolyte thickness, R is the resistance of the electrolyte, and A is the area of the blocking electrode) (Gadjourova et al., 2001; Dyre et al., 2009; Hu et al., 2015).

To understand the effect of LLZO within the electrolyte membrane of lithium symmetric cells with lithium metal as the negative electrode and PLL, the charge–discharge voltage profile of the electrolyte within the Li/PLL/Li cell was measured under various current densities of 0.1, 0.3, and 0.5 mA cm^−2^ with a charge and discharge time of 30 min per cycle. For each condition, the cell was tested for 50 cycles (50 h) to verify the stability of the electrolyte with lithium metal. The LANHE CT2001A battery testing system was used to investigate the long cycle stability of the electrolyte with the lithium metal electrode.

### Battery Performance in Full-Cell Testing With LiFePO_4_ as Cathode Materials

All-solid-state batteries consisting of LiFePO_4_ (LFP) as the cathode active material, lithium as anode and with the electrolyte part being a membrane, with no liquid electrolyte or separator used, were fabricated in an glove box filled with argon. The cathode-basis LFP was prepared by mixing LiFePO_4_, super-P, and PEO-LiTFSI (molar ratio of EO: Li = 8:1) as a binder, with a weight ratio of 8:1:1. This cathode was used to fabricate all-solid-state batteries operating at 60°C with various SPEs, including PL and PLL, to compare the electrochemical properties of the electrolyte and determine the effect of LLZO within the electrolyte. The comparison was carried out with a constant current of 50 mA g^−1^ and the same LFP cathode. Moreover, the LFP/PLL/Li battery was tested with the C-rate at various currents from 10 to 200 mA g^−1^ and back to 10 mA g^−1^. Charge and discharge tests of the all-solid-state lithium batteries were performed using a battery testing instrument (CT2001A, LANHE). The active material mass loading of LFP was ~2 mg cm^−2^, and the lithium metal chip as anode (MTI Korea) was used with the thickness of 450 μm and the diameter of 16.0 mm.

## Results and Discussion

**Figure 1:**
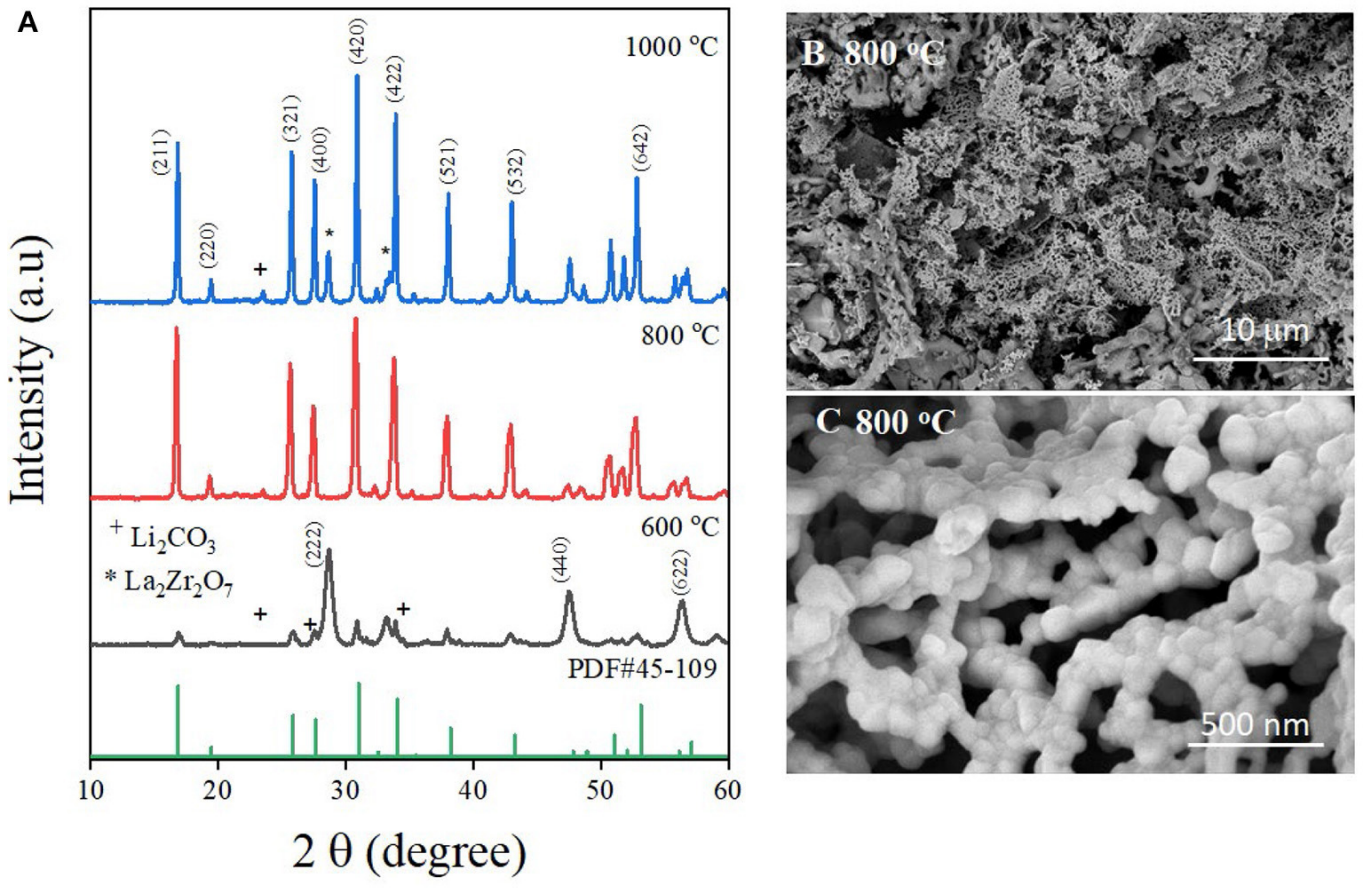
**(A)** Comparison of X-ray diffraction patterns of LLZO synthesized at various temperatures. SEM images of LLZO nanoparticles synthesized at 800°C at **(B)** low magnification, and **(C)** high magnification.

The XRD patterns of LLZO fine powder synthesized at different temperatures are depicted in Figure 1A. The diffraction peaks of the LLZO powder synthesized at 600°C correspond to the mixture of La_2_Zr_2_O_7_ (JCPDS 73-0444) and Li_2_CO_3_ (PDF#22-141). The results could be explained by the incomplete decomposition reaction of the substances. Even though the decomposition temperature of Li_2_CO_3_ is 600°C, this is only the starting point of the decomposition reaction. Therefore, Li_2_CO_3_ could not be decomposed completely. After elevating the temperature to 800°C, the decomposition reaction of Li_2_CO_3_ was boosted, leading to the complete decomposition of the substances and the formation of LLZO. The XRD pattern of the synthesized LLZO nanoparticle at 800°C is similar to that of the reference LLZO (PDF#45-0109) with all reflections corresponding to a cubic *Ia3d* symmetry (Sun et al., 2019). The result indicates that cubic phase LLZO has been formed and no diffraction peak characteristic of the tetragonal phase LLZO has been detected. It should be noted that the cubic phase LLZO shows better ionic conductivity than the tetragonal phase LLZO. The particle size of as-synthesized LLZO can be calculated using the Scherrer equation (Scherrer, 1912):

$$\begin{array}{l} \text{D}=\frac{\text{K}\lambda }{\beta cos\theta } \end{array}$$

where D is the average particle size, K is the Scherrer constant (for a spherical crystallite, K = 0.89), λ is the incident X-ray wavelength, β is the full width at the half-maximum of the diffraction peak, and θ is the angle at the diffraction peak. Using this equation, the particle size is calculated as ~30.9 nm. With the temperature increased to 1,000°C, in addition to the characteristic peaks of cubic phase LLZO, some impurities of La_2_Zr_2_O_7_ were also detected. This could be explained by the loss of lithium by evaporation of the alkali metal at elevated temperatures. After evaporation occurs, lithium is released from the structure, leading to the transformation from LLZO to La_2_Zr_2_O_7_. To understand the morphology of the LLZO nanoparticles, the SEM images of the material are shown in Figures 1B,C. At low magnification, LLZO bulk particles can be clearly observed as a garnet structure, where the particles connect to form a network. Figure 1C shows LLZO nanoparticles at high magnification, where the size and shape of LLZO particles can be seen clearly. As shown in the figure, each small LLZO nanoparticle consolidates to form an LLZO garnet network that can enhance the ionic conductivity of the bulk material. The LLZO particles appear to have a spherical shape with the size estimated at 35 nm, which is consistent with the particle size calculated from XRD data. Moreover, the XRD data and SEM images of LLZO demonstrate that the formation of garnet type cubic LLZO was achieved using the sol-gel method at a low temperature of ~800°C. In addition, the size of the LLZO particles is in the range proposed by several researchers for the cubic phase of LLZO. At elevated temperatures, the formation of agglomerated particles of micron size could be the result of transformation from the cubic phase into the tetragonal phase of LLZO. This phenomenon would result in a decline in the ionic conductivity of the bulk material.

**Figure 2:**
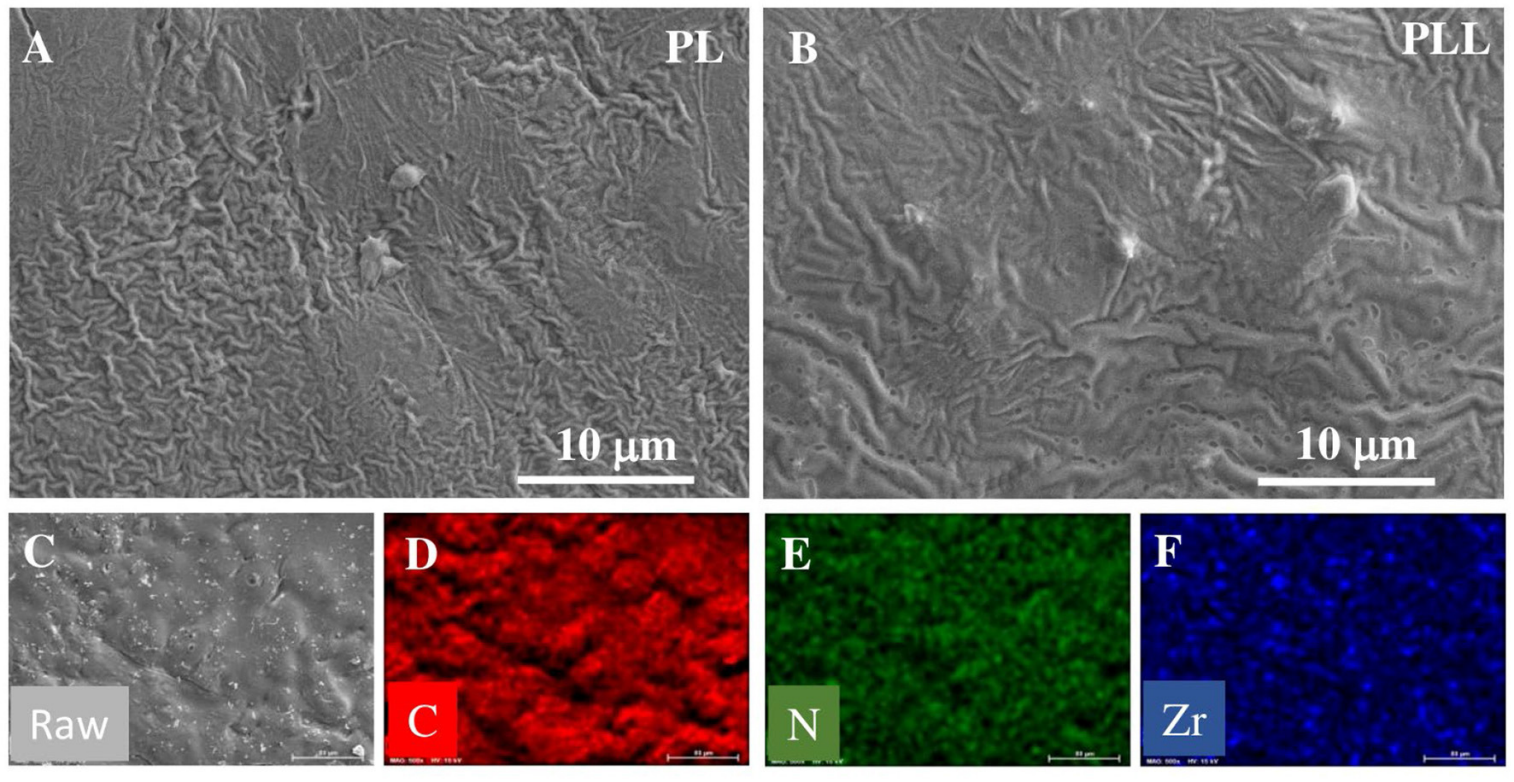
SEM images of solid polymer membrane: **(A)** PL electrolyte, and **(B)** PLL electrolyte. EDX mapping of PLL electrolyte: **(C)** Electrolyte membrane, **(D)** C element, **(E)** N element, and **(F)** Zr element.

The freestanding film of solid polymer electrolyte were fabricated after completely drying in the vacuum oven, maintaining robust structure of freestanding film at high temperature (60°C). Supplementary Figures 1, 2 show the stable film of solid polymer electrolyte at high temperature. The surface morphologies of different types of electrolytes were studied using SEM, and the resulting images are shown in Figures 2A,B. Figure 2A shows the SEM images of the PL electrolyte; the surface of the electrolyte membrane is not smooth, with several wrinkles. This is ascribed to the natural crystallinity of the PEO matrix polymer, which could be the reason for the ionic conductivity of this electrolyte. On the contrary, the PLL electrolyte possesses fewer wrinkles than the PL electrolyte, which is the result of the incorporation of LLZO into the electrolyte. In addition, some large particles and holes could be observed, which is the consequence of the agglomeration of LLZO in the dispersion process. Based on this surface observation, the use of LLZO nanoparticles as a filler in the electrolyte is expected to increase the ionic conductivity and mechanical properties of the solid polymer membrane. These results are already promising for the electrochemical performance of the battery with the PLL electrolyte. To investigate the distribution of LLZO within the electrolyte, energy dispersive X-ray spectroscopy (EDX) mapping was employed, and the detailed elemental mapping of the PLL membrane is shown in Figures 2C–F. The EDX mapping of N corresponds to the nitrogen in the structure of LiTFSI and Pyr_1,3_TFSI. The EDX mapping of C represents the carbon in PEO and Pyr_1,3_TFSI. By comparing the N and C mappings, it can be concluded that the salt and ionic liquid are homogeneously distributed within the PEO matrix. In addition, the EDX mapping of Zr, which corresponds to zirconium in LLZO, indicates that LLZO is also present throughout the sample. Moreover, it demonstrates that the overall morphology of the electrolyte membrane and LLZO nanoparticles are well-dispersed in the SPE membrane.

**Figure 3:**
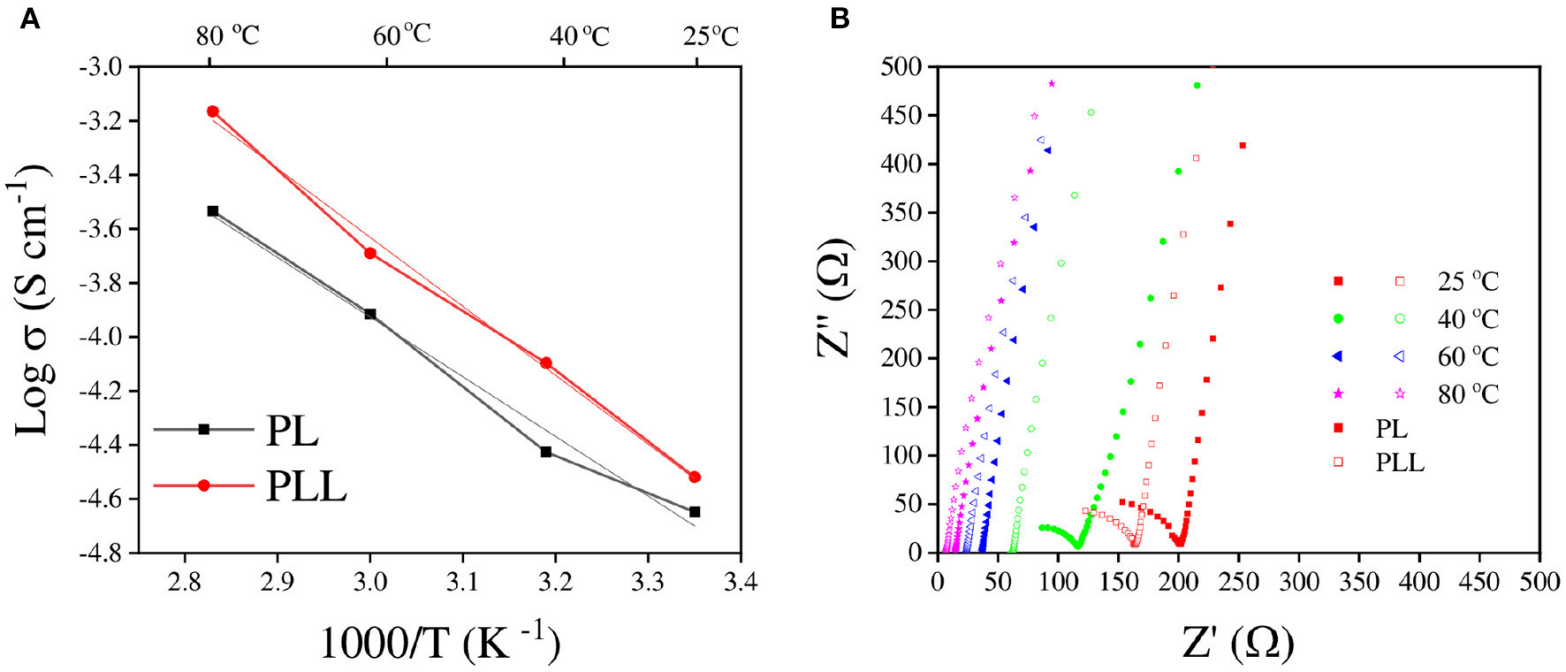
**(A)** Ionic conductivity plots of the PL and PLL solid polymer electrolytes. **(B)** Nyquist plots of the PL and PLL solid polymer electrolytes performed at various temperatures.

The ionic conductivity is one of the most imperative indicators for estimating the ability of the electrolyte to be used in electrochemical applications as an ion transferring component. The ionic conductivity of the electrolytes could not be evaluated directly, however, it can be calculated from the resistance of the electrolyte. In this study, the electrolyte was “sandwiched” between two stainless steel disks to measure its resistance using electrochemical impedance spectroscopy (EIS). The resistance indicates the extent to which the electrolyte blocks ions from moving through it. Therefore, a lower resistance means better ionic conductivity. The ionic conductivity and Nyquist plot of the electrolyte at different temperatures are shown in Figure 3. The values of ionic conductivity of PL and PLL at 25°C were 2.24 × 10^−5^ and 3.02 × 10^−5^ S cm^−1^, respectively (Figure 3A). The addition of 10 wt% garnet cubic phase LLZO induced ~40% increase in the ionic conductivity at 25°C. When the temperature was increased to 60°C, the ionic conductivity of the SPEs increased to 1.2 × 10^−4^ and 2.0 × 10^−4^ S cm^−1^ for the PL and PLL electrolytes, respectively, which means that the ionic conductivity of PLL was nearly double compared to that of PL. Furthermore, when the temperature was increased, the gaps between the ionic conductivities of these electrolytes increased too. It can be inferred that the presence of LLZO was able to stabilize the electrolyte at high temperatures, which ensures the performance of the battery under these conditions. The details of the resistance and ionic conductivity of the solid polymer membrane are shown in Table 1. Supplementary Table 2 illustrates the activation energy of the PEO electrolyte with and without LLZO. It can be observed that the activation energy can decrease when LLZO is present in the PEO electrolyte. When the ionic conductivities of the PL and PLL are fitted to the Arrhenius equation (Figure 2A), both of them show an approximately linear correlation with temperature, where the linear correlation coefficients of PL and PLL are 0.987 and 0.995, respectively. It can be estimated that the activation energies (Ea) of the lithium-ion migration of PL and PLL are 0.35 and 0.25 eV, respectively. A reduced Ea value further implies that quicker liquid-phase lithium-ion migration occurred after the addition of LLZO in PL. Moreover, since LLZO has good thermal stability, its addition would also enhance the thermal stability of the electrolyte, in contrast to organic carbonate additives. The high thermal stability can perfectly meet the safety requirements for lithium metal batteries.

**Table 1:** Resistance and ionic conductivities of the PL and PLL electrolytes at different temperatures.

**Temperature (°C)**		**25**		**40**		**60**		**80**	
**Electrolyte**	**Thickness (μm)**	**R**	**σ**	**R**	**σ**	**R**	**σ**	**R**	**σ**
PL	90	200.2	2.25 × 10^−5^	120.2	3.74 × 10^−5^	37	1.21 × 10^−4^	15.4	2.92 × 10^−4^
PLL	100	165.3	3.02 × 10^−5^	62.5	8 × 10^−5^	24.5	2.04 × 10^−4^	7.3	6.85 × 10^−4^

To quantify Lithium ion conductivity, the symmertric Li/SPE/Li (lithium metal as electrode) was test with the same measurement method of previous research (Ohta et al., 2011). The result of lithium ion conductivity was present in Supplementary Table 3, Supplementary Figure 3. In this result, lithium ion of PLL electrolyte is 2 times higher than that of PL electrolyte, presenting the improved Li ion conductivity by the addition of LLZO. In the Supplementary Figure 3, we also caculated the lithium activation energy of solid polymer electrolyte to compare the Li ion mobility. The activation energy of PL is higher (0.26 eV) than that of PLL (0.13 eV) which the solid polymer electrolyte without LLZO requires more energy for lithium ion mobility in the SPE. This result clearly indicate the correlation between lithium ion conductivity and lithium ion mobility in the SPE.

The Linear sweep voltammetry of the solid polymer electrolyte was shown in Supplementary Figure 4, in which the reference electrode is lithium metal, and working electrode is stainless steel. The cell was tested with the potential range of 3 V ~ 6 V at a scan rate of 1 mV s^−1^. It showed stably maintained current until 5.0 V to both of cells. From the linear plot result, the oxidation peak of PLL and PL presents around 5.3 and 5.1 V, respectively. It indicates the LLZO deternined more stable electrochemical reaction, resulting in the stability of solid polymer electrolyte at high voltage operation of all-solid-state batteries (Li et al., 2015; Homann et al., 2020).

**Figure 4:**
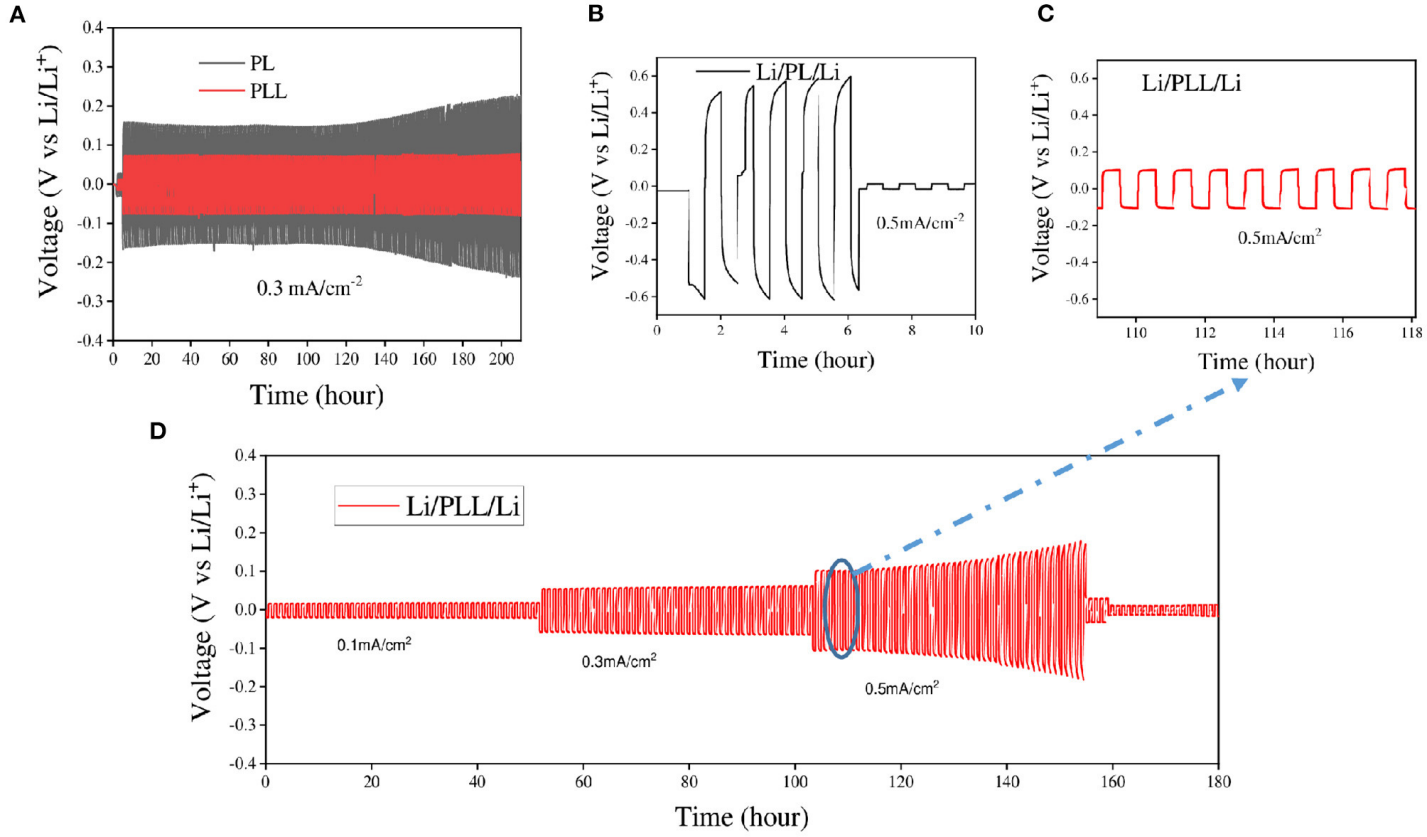
Comparison of Li/PL/Li with Li/PLL/Li in lithium stripping/plating tests at various current densities **(A)** at 0.3 mA cm^−2^ and 60°C, and **(B,C)** at 0.5 mA cm^−2^ and 60°C. **(D)** Lithium stripping/plating profiles of the PLL electrolyte at various current densities.

Following the electrochemical stability test, the application of LLZO to suppress lithium dendrite growth in the electrode was confirmed with the lithium stripping/plating test. The coin cells with symmetric lithium metal as electrodes and PL or PLL as electrolytes were charged and discharged at various current densities ranging from 0.1 to 0.5 mA cm^−2^ at 60°C. Figure 4 shows the galvanostatic voltage profiles of lithium stripping/plating with PL or PLL electrolyte membranes to investigate the reaction between the electrolytes and lithium metal. As shown in Figure 4A, at a current density of 0.3 mA cm^−2^, the cell with the PLL electrolyte shows stable voltage windows ranging from −0.07 to 0.07 V for over 200 cycles (over 200 h). The presence of LLZO within the electrolyte results in a stable surface resistance between the electrolyte and the surface of the lithium metal. LLZO leads to the homogeneous distribution of lithium on the lithium metal surface in the plating reaction. In addition, LLZO exhibits good mechanical strength that blocks the dendrites from piercing through the PEO matrix. It should be noted that when the dendrites pierce into the electrolyte, this results in an increase in the electrolyte resistance and reduces the ionic conductivity. When the dendrites pierce through the electrolyte and reach the surface of the cathode, this leads to a short circuit within the cell, followed by thermal runaway. In the case of the PL electrolyte, without LLZO, the higher interfacial resistance results in higher voltage windows as required for the stripping/plating reaction. For the first 120 cycles, the voltage windows range from −1.6 to 1.6 V which is more than double compared to that of the cell with the PLL electrolyte. Moreover, after 120 cycles, the reaction within this cell became critical, which is inferred from the rapid increase in the voltage windows. This signals the formation of dendrites and the piercing of dendrites into the electrolyte. If the cycling continued for longer, a short circuit would occur after a few more cycles. The results reveal that the PL electrolyte is weak and unstable even at a middle-strength current density of only 0.3 mA cm^−2^. Even when the cell with the PL electrolyte was tested at a current density of 0.5 mA cm^−2^, a high voltage window of −0.6 to 0.6 V was observed followed by a short circuit after only five cycles (Figure 4B). In the case of the PLL electrolyte, the cell was more stable with small voltage windows from −0.1 to 0.1 V (Figure 4C). The limitation on the current density that can be applied to a battery is normally studied using the rate type of the current density applied to the cell. Figure 4D presents the rate stability of the PLL electrolyte at current densities of 0.1, 0.3, and 0.5 mA cm^−2^ for 50 cycles for each current density. The battery shows stable cycles and voltage windows below 0.1 and 0.3 mA cm^−2^ of current density. However, when subjected to 0.5 mA cm^−2^, the cell was only stable for 30 cycles before the increase of the voltage windows followed by the short circuit of the battery. This result reveals that the battery with the PLL membrane should be operated under a current density lower than 0.5 mA cm^−2^. To improve the working current density for higher energy density batteries using this electrolyte, a further strategy should be proposed.

**Figure 5:**
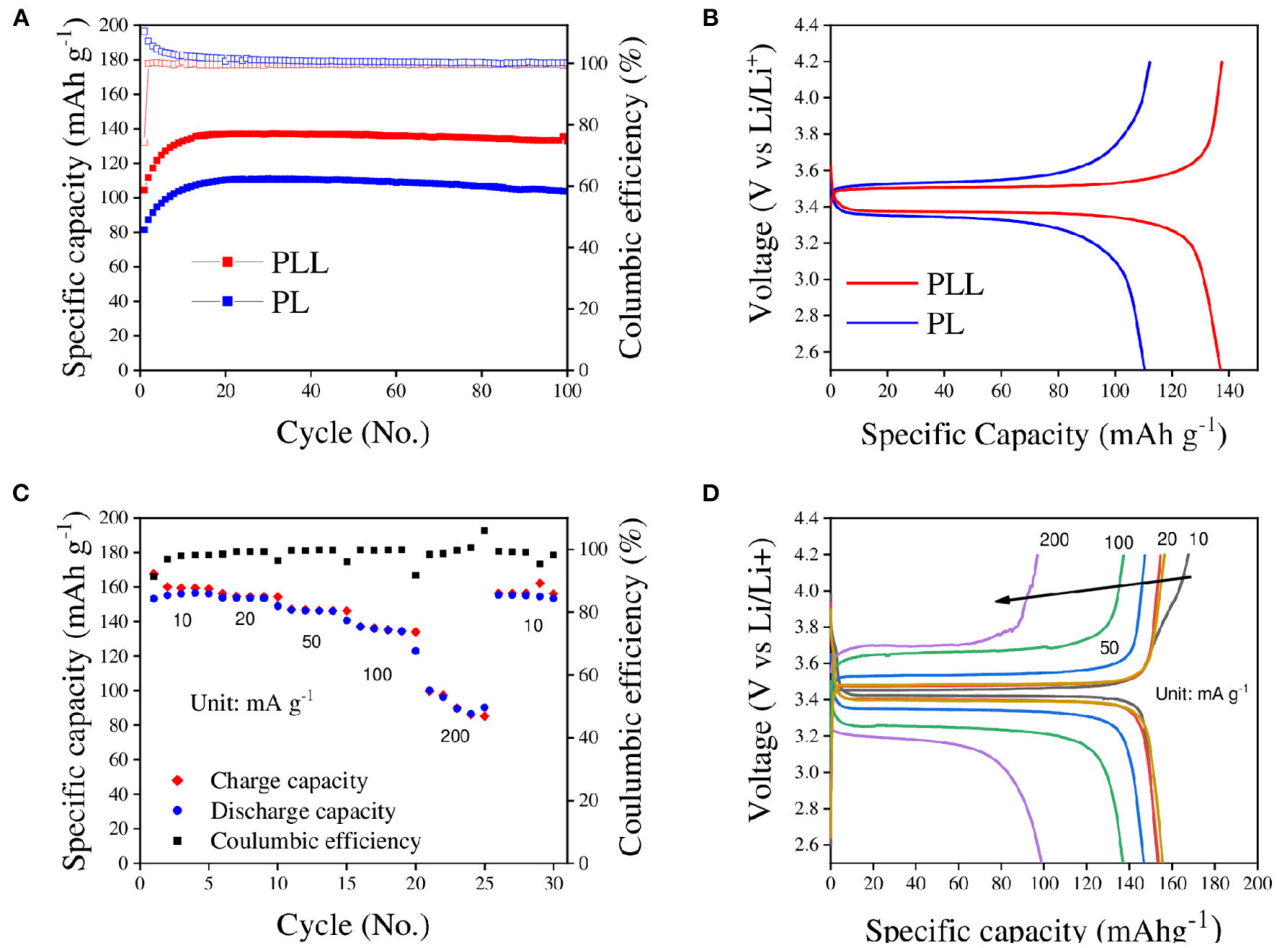
**(A)** Long cycle performances of the PLL and PL electrolytes in full-cell tests (LiFePO_4_/PLL/Li and LiFePO_4_/PL/Li) at 50 mA g^−1^ current density and 60°C for 100 cycles. **(B)** Galvanostatic charge-discharge profiles of the batteries with the PLL and PL electrolytes at the 25th cycle derived from **(A)**. **(C)** Rate capability of the battery with the PLL electrolyte with the current density ranging from 10 to 200 mA g^−1^. **(D)** Galvanostatic charge-discharge profiles of the battery with the PLL electrolytes for the rate capability test at the first cycle of each current density derived from **(C)**.

Having created a stable electrolyte and clarified its impact, the electrochemical performance of the electrolyte for lithium ion batteries was described. Figure 5A shows the long cycle test of the PLL electrolyte determined by electrochemical evaluations of the full-cell at a current density of 50 mA g^−1^. As expected from the tests above, the cell with the PLL electrolyte showed better discharge specific capacity compared to the cell with the PL electrolyte because of its higher ionic conductivity and stability. Both cells showed low initial discharge capacities, and the discharge capacity gradually increased for the first 20 cycles. The maximal discharge capacities reached were 137.4 and 110.9 mAh g^−1^ for the cell with the PLL and PL electrolyte, respectively. It should be noted that, based on several studies, a battery with an SPE requires an amount of time to be activated at high temperature. Through activation, the connection between electrolytes and both electrodes can become stronger. Therefore, the increase in the discharge capacity for both electrolytes could be the result of the activation process. After 100 cycles, the discharge capacities of the cells with the PLL and PL electrolytes remained at 135.3 and 103.7 mAh g^−1^, respectively, corresponding to 98.47 and 93.5% capacity retention. After long cycles, the disadvantage of the PL electrolyte was evident through the fast capacity decay compared to the cell with the PLL electrolyte. Moreover, it is worth noting that by adding only 10% LLZO into the electrolyte, not only was the capacity enhanced by 24% but also the stability against a long cycle test with a lithium metal anode was improved. On the other hand, the galvanostatic charge-discharge profiles of the cells with the PLL and PL electrolytes at the 25th cycle are shown in Figure 5B. Both batteries exhibited only one plateau for each charge or discharge curve corresponding to the oxidation or reduction reaction between LiFePO_4_ and ${\text{FePO}}_{4}^{-}$. The polarization value between the charge and discharge curve of the cell with the PLL electrolyte is 126 mV, which is quite small and consistent with the result for the ionic conductivity. As calculated above, the PLL electrolyte exhibits a high ionic conductivity; therefore, both its intrinsic resistance and interfacial resistance are better than those of the PL electrolyte. This is also consistent with the high polarization value of the cell with the PL electrolyte, which is 161 mV. The lower the ionic conductivity of the electrolyte, the higher the polarization value required. The rate capability of the battery with the PLL electrolyte is presented in Figure 5C. The initial discharge capacities were 153.3, 154, 146.8, 137, and 100.3 mAh g^−1^ obtained at current densities of 10, 20, 50, 100, and 200 mA g^−1^, respectively. The rate ability performance at these current densities reveals excellent capacity even in the high-rate cell test. After changing the current density back to 10 mA g^−1^, the discharge capacity recovered to 155.5 mAh g^−1^, which demonstrates the stability of the battery performance after treatment under high current density. Because of the activation in the first few cycles, the first discharge capacity of the first cycle was 153.3 mAh g^−1^ and then gradually increased to the maximal point of 156.6 mAh g^−1^ at the fourth cycle. Comparing the discharge capacity of the initial phase and the recovery phase at 10 mA g^−1^, the recovery rate was as high as 99.3%. Moreover, the severe decay of the discharge capacity occurs at a high current density of 200 mA g^−1^, which might be due to the current density limitation of the PLL electrolyte. Given that the mass loading of the cathode material was 2 mg cm^−2^, under the effect of 200 mA g^−1^ current density, it can be calculated as 0.4 mA cm^−2^, which is between the stable and unstable value of the current density as obtained from the stripping/plating test. This can be further demonstrated by analyzing the galvanostatic charge-discharge curves derived from the rate capability, which are shown in Figure 5D. The differences in the polarization voltages at 10, 20, 50, and 100 mA g^−1^ current densities are small, implying that the battery is stable at these current densities. When the current density is changed to 200 mA g^−1^, the polarization voltage increases significantly, which means that the resistance is high and threatens the safety of the battery. If the cycling under 200 mA g^−1^ current density with a high mass loading electrode is longer, the cell might suffer from a short circuit.

## Conclusions

In summary, cubic phase LLZO with a garnet structure was successfully prepared by the sol-gel method. Cubic LLZO was prepared at a low temperature of 800°C without any impurities and formed a homogeneous structure. The addition of LLZO in the solid polymer electrolyte led to the improvement of the ionic conductivity and the activation energy in comparison with the electrolyte without LLZO. Moreover, the addition of LLZO was able to improve the battery performance and diminish the lithium dendrite formation during cycling. As a result, the battery with the PLL electrolyte exhibited superior performance with a high specific capacity of 137.4 mAh g^−1^ at the maximal point and 135.3 mAh g^−1^ after 100 cycles with 98.47% capacity retention at 50 mA g^−1^ current density. Moreover, the specific discharge capacities were 137 and 100.8 mAh g^−1^ at 100 and 200 mA g^−1^ current densities, respectively. Thus, the batteries were stable for long cycles in all-solid-state applications at high temperatures and fast charging systems.

## Data Availability Statement

The original contributions presented in the study are included in the article/Supplementary Material, further inquiries can be directed to the corresponding author/s.

## Author Contributions

All authors listed have made a substantial, direct and intellectual contribution to the work, and approved it for publication.

## Conflict of Interest

The authors declare that the research was conducted in the absence of any commercial or financial relationships that could be construed as a potential conflict of interest.
