# Supplementary material for: Li7La3Zr2O12 Garnet Solid Polymer Electrolyte for Highly Stable All-Solid-State Batteries
Source: Front Chem. 2021 Jan 18;8:619832. doi: 10.3389/fchem.2020.619832 (PMC7847977; doi:10.3389/fchem.2020.619832)
Supplement: Supplementary file 1 [file Data_Sheet_1.docx]

SUPPORTING INFORMATION

Li_7_La_3_Zr_2_O_12_ garnet solid polymer electrolyte for highly stable all-solid-state batteries

Quoc Hung Nguyen^1^ ^†^, Van Tung Luu^1^ ^†^, Hoang Long Nguyen^1^, Young-Woo Lee^1^, Younghyun Cho^1^, Se Young Kim^2^, Yun-Seok Jun^3,^*, Wook Ahn^1,^*

^1^Department of Energy Systems Engineering, Soonchunhyang University, 22 Soonchunhyang-ro, Shinchang-myeon, Asan-si, Chungcheongnam-do 31538, Korea.
E-mail: [wahn21@sch.ac.kr](mailto:wahn21@sch.ac.kr)

^2^Department of Chemistry, University of Waterloo, 200 University Avenue West, Waterloo, Ontario, N2L3G1 Canada

^3^Microcellular Plastics Manufacturing Laboratory, Department of Mechanical and Industrial Engineering, University of Toronto, 5 King's College Road, Toronto, Ontario, M5S 3G8, Canada

*** Correspondence:**Corresponding Author
E-mail: [ysjun@mie.utoronto.ca](mailto:ysjun@mie.utoronto.ca), [wahn21@sch.ac.kr](mailto:wahn21@sch.ac.kr)

Keywords: all-solid-state batteries, cubic garnet llzo, ionic-liquid, lithium-ion battery, lithium iron phosphate, solid polymer electrolytes, suppress lithium dendrite


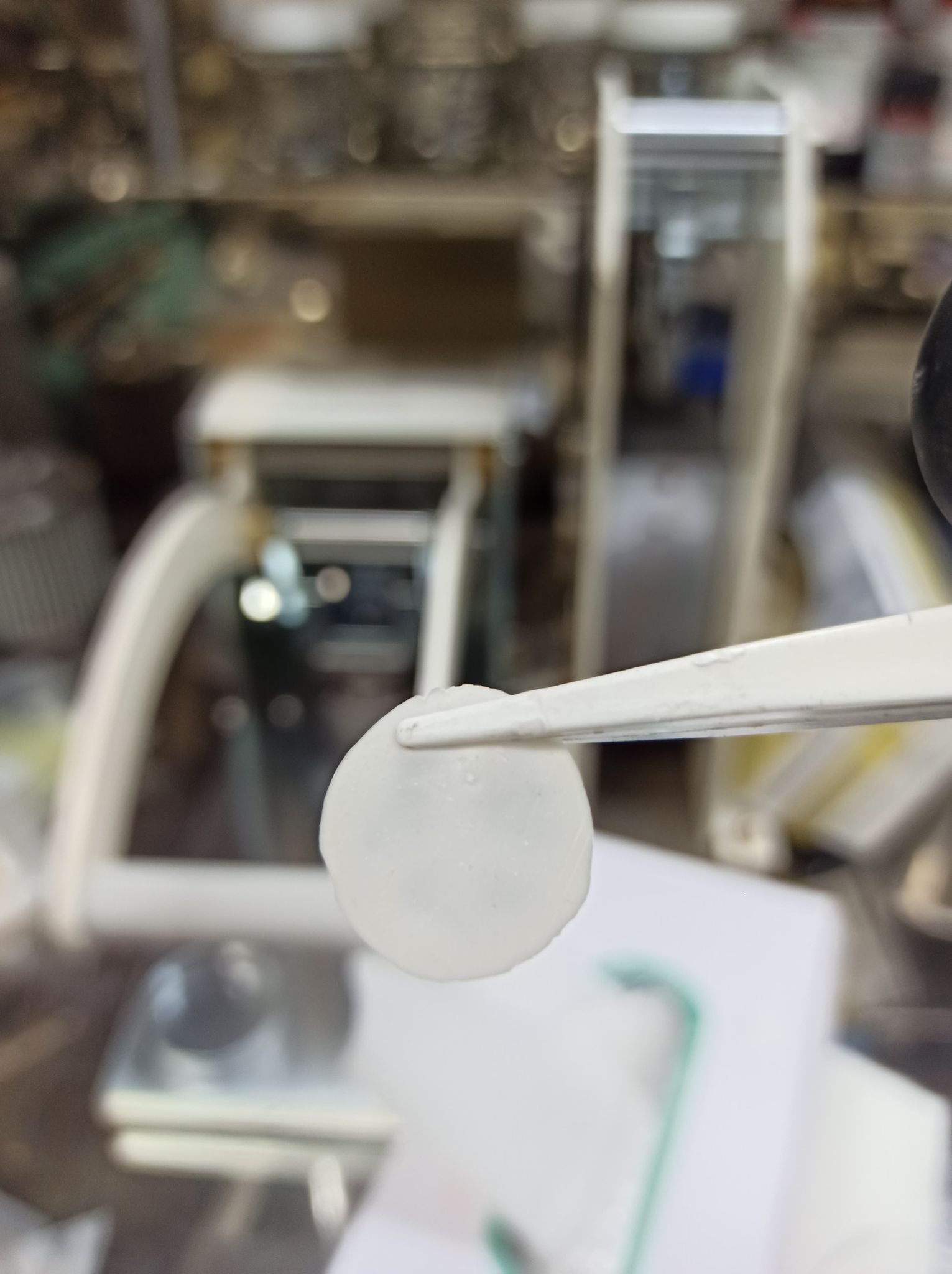

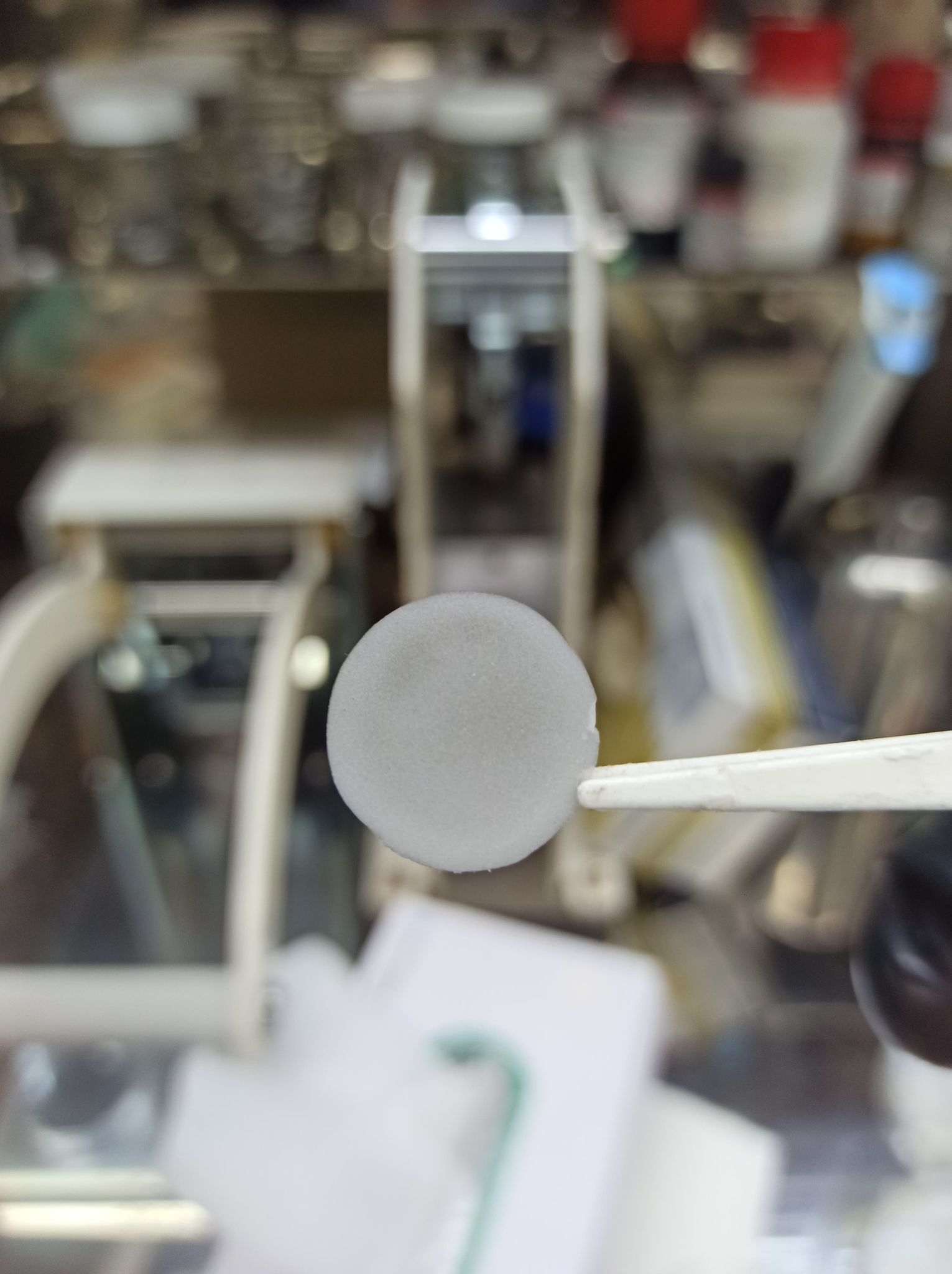


PL

PLL

**FIGURE S1 |** Solid polymer membrane at room temperature after drying. This membrane so the structure like the solid-state such as Fuiru Ma and co-worker also present about solid polymer electrolyte based on polymerized ionic liquid for all-solid-state-batteries with high thermal stability (Ma et al., 2019).


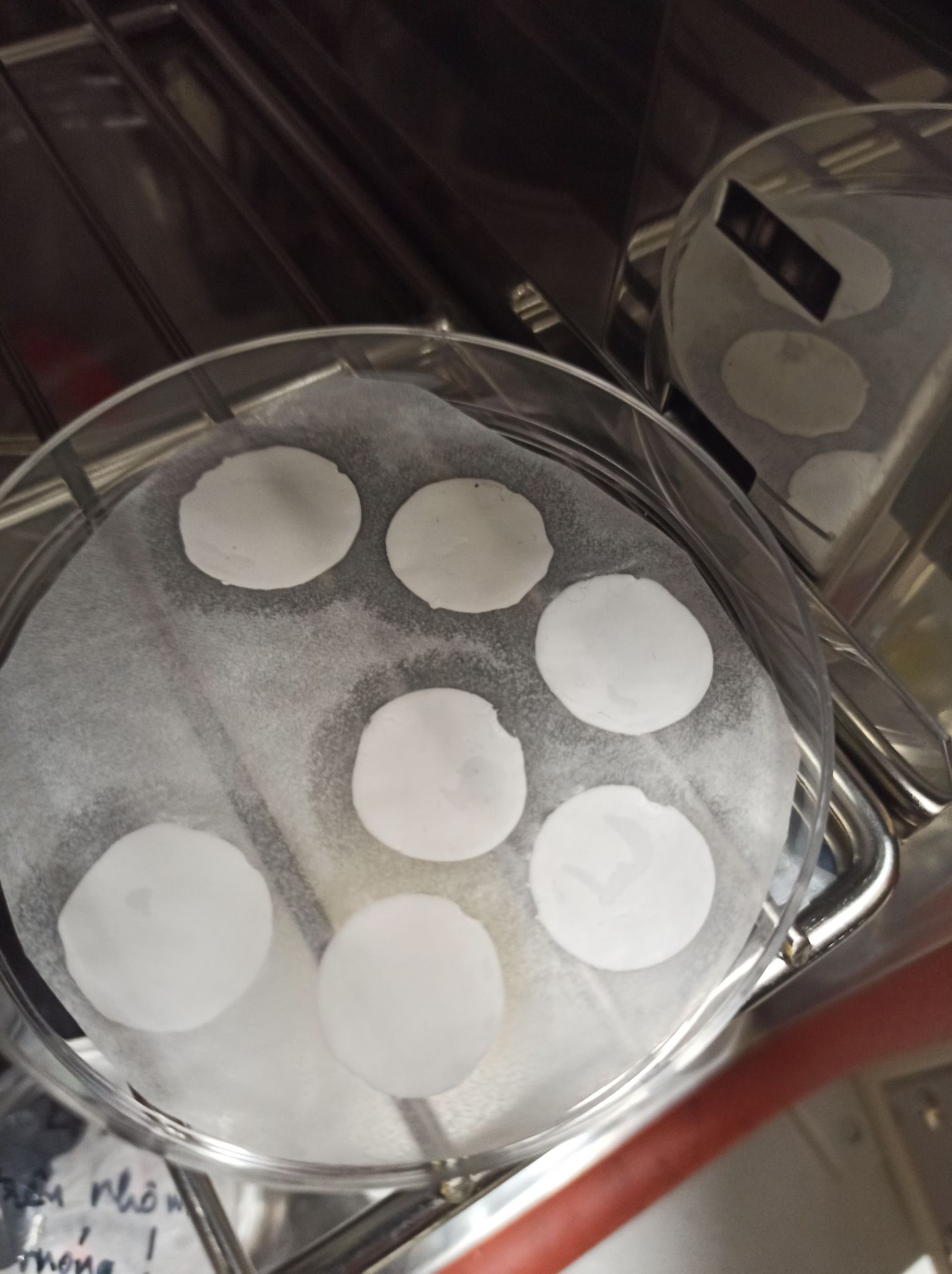


**FIGURE S2 |** The stability of solid polymer membrane at high temperature (60 ^o^C). The membrane keep the structrure and did not change the quasi-solid state.


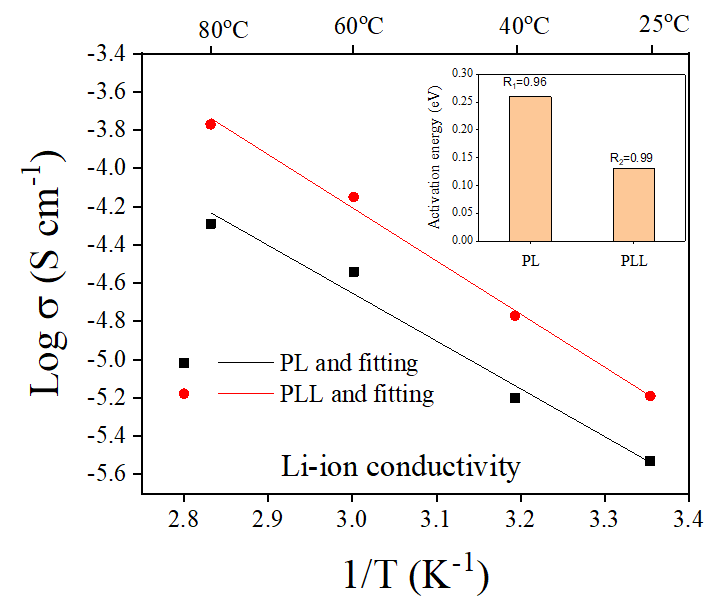


**FIGURE S3 |** Plot of lithium ionic conductivity of solid polymer electrolyte membrane.

**FIGURE S4 |** LSV of solid polymer electrolyte at 60 ^o^C

|  | | 600^o^C | | | | | | 800^o^C | | | | | | 1000^o^C | | | | | |
| --- | --- | --- | --- | --- | --- | --- | --- | --- | --- | --- | --- | --- | --- | --- | --- | --- | --- | --- | --- |
| No. | | 2θ | | FWHM | | D | | 2θ | | FWHM | | D | | 2θ | | FWHM | | D | |
| 1 | | 28.66 | | 0.68 | | 12 | | 16.72 | | 0.22 | | 35.88 | | 16.81 | | 0.18 | | 43.46 | |
| 2 | | 47.53 | | 0.73 | | 11.94 | | 25.64 | | 0.28 | | 29.49 | | 25.75 | | 0.2 | | 41.55 | |
| 3 | | 56.34 | | 0.77 | | 11.74 | | 27.45 | | 0.3 | | 27.35 | | 27.56 | | 0.2 | | 41.46 | |
| 4 | | - | | - | | - | | 30.75 | | 0.32 | | 25.91 | | 28.63 | | 0.26 | | 31.85 | |
| 5 | | - | | - | | - | | 33.76 | | 0.35 | | 23.8 | | 30.88 | | 0.21 | | 39.85 | |
| 6 | | - | | - | | - | | 37.89 | | 0.36 | | 23.34 | | 33.9 | | 0.22 | | 38.62 | |
| 7 | | - | | - | | - | | 42.86 | | 0.4 | | 21.22 | | 38.04 | | 0.22 | | 38.98 | |
| 8 | | - | | - | | - | | - | | - | | - | | 43.02 | | 0.23 | | 37.33 | |
| **D _average_** | **-** | | **-** | | **11.9** | | **-** | | **-** | | **30.9** | | **-** | | **-** | | **42.16** | |  |

**TABLE S1 |** Particle size of LLZO nanoparticles synthesized at different temperatures (calculated from XRD data using Scherrer equation) (Sakakibara et al, 2019).

| Electrolyte | Slope (K) | Activation Energy (E_a_) |
| --- | --- | --- |
| PL | -0.17631 | 0.3498994 |
| PLL | -0.12724 | 0.2525166 |

**TABLE S2 |** The activation energy of PL and PLL electrolyte calculated using Arrhenius plot.

Following the Arrhenius equation,

$$log\sigma=logA- \frac{2.303Ea}{RT}$$

A is referred to the pre-exponential factor, E_a_ represents the activation energy, R represents the gas

Constant and T is the absolute temperature. Thus,

$$log\sigma=logA- \frac{2.303Ea}{1000R}\frac{1000}{T}$$

We call X as 1000/T, Y is logσ, we have:

$$K=- \frac{2.303Ea}{R}$$

By taking the slope K from the Arrhenius plot and using the above equation, E_a_ could be calculated.

| Temperature (^o^C) | | 25 | | 40 | | 60 | | 80 | |
| --- | --- | --- | --- | --- | --- | --- | --- | --- | --- |
| Electrolyte | Thickness (µm) | R | σ | R | σ | R | σ | R | σ |
| PL | 100 | 1698.0 | 2.95  ×10^-6^ | 799.3 | 6.26  ×10^-6^ | 175.9 | 2.84  ×10^-5^ | 97.8 | 5.15  ×10^-5^ |
| PLL | 100 | 788.6 | 6.34  ×10^-6^ | 294.7 | 1.70  ×10^-5^ | 71.2 | 7.02  ×10^-5^ | 29.4 | 1.70  ×10^-4^ |

**TABLE S3|** Detail of Li-ionic conductivity of polymer electrolyte.

Ma, F., Zhang, Z., Yan, W., Ma, X., Sun, D., Jin, Y., et al. (2019). Solid polymer electrolyte based on polymerized ionic liquid for high performance all-solid-state lithium-ion batteries. *ACS Sustainable Chem. Eng.* 7(5)**,** 4675-4683.
